# Fatal Case of Gout

**Published:** 1831

**Authors:** 


XLVII.

Fatal Case of Gout. By M. Sauvé.

This case is related in the Annales de la Médecine Physiologique, for April, 1831. The patient, æt. 62, was admitted into the Hospital of the Val-de-Grace, in 1831. He had suffered much from gout previously. The articulations of the fingers and toes were swollen, and misshapen, the appetite great, the corpulency considerable. Some-time after the patient's admittance a gouty abscess formed in the right foot; it was opened, and the bone was found dead. For some months there was little else complained of excepting pain in the affected foot, but at length a "gastro-enterite" was set up, the liver became implicated, and jaundice was established. In spite of all the treatment employed the patient died.

*Sectio Cadaveris.* All the articulations were filled with a soft whitish matter, resembling in appearance cream-cheese, which extended into the spongy substance of the bones, leaving no trace of synovial membrane or cartilage. The compact substance of the bones was brownish in colour, and the periosteum was easily detached. The muscles were of a pale red colour, the cellular tissue indurated. In the right leg were several calcareous concretions external to the periosteum, and even on the fasciæ. In the knee-joint the synovial membrane and its appendages were reddened and very soft, and a large abscess extended from within the joint upwards, between the femur and the muscles surrounding the bone, as high as the hip-joint. Of this there had been no symptom during life. On the left tibia were several deposits of cretaceous matter. A yellow very viscid liquid was found in the left knee-joint, and the condyles were covered with a radiated chalky deposit. The same chalky concretions were observed in the joints of the upper extremities, and the sheath of the tendons and the ligaments were infiltrated with the same. The heart was almost buried in fat. The mucous membrane of the stomach was of a bright red towards the cardia, of a deeper dye towards the pylorus, which was indurated and thickened. The mucous membrane of the duodenum was softened, almost ulcerated, and the marks of inflammation extended down the intestinal tube, decreasing as it descended.

---

